# Apathy in Alzheimer’s disease: Contribution to a clinical view on progression of dementia

**DOI:** 10.1590/S1980-57642010DN40300007

**Published:** 2010

**Authors:** Florindo Stella, Larissa Pires de Andrade, Thays Martins Vital, Flávia Gomes de Melo Coelho, Carla Manuela Crispim Nascimento, Salma Stephany Soleman Hernández

**Affiliations:** 1 UNESP, Universidade Estadual Paulista, Biosciences Institute, Campus of Rio Claro, Aging and Physical Activity Laboratory, São Paulo SP, Brazil.; 2 Geriatric Psychiatry Clinic, State University of Campinas, CRUESP Cooperation Program, Campinas SP, Brazil.

**Keywords:** apathy, Alzheimer’s disease, disease progression

## Abstract

In addition to cognitive impairment, apathy is increasingly recognized as an important neuropsychiatric syndrome in Alzheimer’s disease (AD).

**Aims:**

To identify the relationship between dementia severity and apathy levels, and to discuss the association of this condition with other psychopathological manifestations in AD patients.

**Methods:**

This study involved 15 AD patients (mean age: 77 years; schooling: 4.9 years), with mild, moderate and severe dementia, living in Rio Claro SP, Brazil. Procedures included evaluation of cognitive status by the Mini-Mental State Examination, Clinical Dementia Rating, and Global Deterioration Scale. Apathy syndrome was assessed by the Apathy Evaluation Scale and Neuropsychiatric Inventory (NPI-apathy domain). Other psychopathological manifestations such as depression were also considered.

**Results:**

Patients with more severe dementia presented higher levels of apathy, reinforcing the hypothesis that apathy severity aggravates as the disease progresses. Using the Spearman coefficient correlation an association was identified between the MMSE and Apathy Evaluation Scale (r=0.63; p=0.01), and also between the MMSE and NPI-apathy domain (r=0.81; p=0.01). Associations were also found between the Global Deterioration Scale and Apathy Evaluation Scale (r=0.58; p=0.02), and between the Global Deterioration Scale and NPI-apathy domain (r=0.81; p=0.01).

**Conclusions:**

Apathy is a distinct syndrome among patients with AD and increases with global deterioration.

## Abstract (pt)

A apatia tem sido reconhecida como uma importante síndrome na doença de Alzheimer (DA), além do comprometimento cognitivo.

**Objetivo:**

Avaliar a relação entre gravidade da demência e níveis de apatia e discutir a associação de apatia com outras manifestações psicopatológicas em pacientes com DA.

**Sujeitos:**

15 pacientes com DA com demência leve, moderada e avançada, residentes em Rio Claro SP, Brasil.

**Métodos:**

Avaliação do status cognitivo por meio do Mini-Exame do Estado Mental, Escore Clínico de Demência e Escala de Deterioração Global. A avaliação da apatia incluiu a Escala de Avaliação de Apatia e o Inventário Neuropsiquiátrico (NPI-domínio apatia). Depressão também foi aferida.

**Resultados:**

Os pacientes com demência avançada apresentaram níveis mais elevados de apatia, o que reforça a hipótese de que ela se agrava na medida em que a doença progride. Por meio do coeficiente de correlação de Spearman, verificou-se associação do Mini-Exame do Estado Mental com a Escala de Avaliação de Apatia (r=0,63; p=0,01) e com o NPI-domínio apatia (r=0,81; p=0,01); também se constatou associação da Escala de Deterioração Global com a Escala de Avaliação de Apatia (r=0,58; p=0,02) e com o NPI-domínio apatia (r=0,81; p=0,01).

**Conclusões:**

A apatia é uma síndrome distinta em pacientes com DA e que se agrava com a deterioração global imposta pela doença.

Although cognitive decline constitutes the clinical hallmark of Alzheimer’s disease, neuropsychiatric symptoms and functional impairments have been strongly considered for its diagnosis, and represent the major psychopathological features of patient suffering and caregiver burden.^1^ As the disease progresses, behavioral disturbances become more frequent and significantly associated with functional impairments,^1^ contributing to a shorter time to institutionalization.^2^ These conditions increase numbers of hospitalizations and emergency room visits, cause excess disability aggravating the decline of daily functioning, and markedly diminish the quality of life of patients and caregivers.^3^

Apathy is a neuropsychiatric syndrome frequently observed in Alzheimer’s disease. Aiming to identify neuropsychiatric syndromes in dementia, Aalten et al.^4^ analyzed cross-sectional studies supported by the European Alzheimer’s Disease Consortium, using the Neuropsychiatric Inventory.^5^ The authors described four clinical factors based on the sum of NPI scores. Apathy was the most common syndrome (65% of scores), followed by hyperactivity (64%), affective disorders (59%) and psychotic syndromes (38%). These percentages could be explained considering that patients presented symptoms of one or more syndromes.^4^

Apathy is increasingly recognized and accepted as an important syndrome present in different neuropsychiatric conditions such as Alzheimer’s disease. As proposed during a consensus meeting coordinated by Robert et al.,^6^ apathy is a persistent motivation disorder whose diagnostic criteria should meet three required domains:

- Diminished motivation;
- Reduced goal-directed behavior, goal-directed cognitive activity, and motivation;
- Functional impairments attributable to apathy.

Robert et al.^6^ established that loss of motivation is a central feature of apathy, representing a core criterion to identify this syndrome. In this context, the patient may present diminished self-initiated behavior and loss of environment-stimulated behavior, as well as reduced interest, for instance in leisure activities, other people or professional targets.

One pertinent question concerns overlap between apathy and depression. Whether apathy and depression constitute one psychopathological syndrome or represent separate conditions remains controversial. According to Aalten et al.,^4^ several symptoms of apathy and depression in part are mixed as although lack of motivation appears in both apathy and depression, unlike depression, the core of apathy is lack of motivation without dysphoria. Robert et al.^6^ affirms that although both conditions share common characteristics, they are distinct and separable by thorough evaluation in dementia and milder cognitive syndromes.

According to Robert et al.,^6^ patients with apathy frequently present functional impairment, and this phenomenon can be attributable to this syndrome, especially to loss of motivation, diminished goal-directed behavior and decreased goal-directed cognitive activity as well as emotional blunting, considered the affective hallmark of apathy. Furthermore, apathy is associated with increase in caregiver burden and suffering.^7^

Despite the high frequency of apathy in neurodegenerative diseases, and increased suffering of patients and caregivers, the findings of non-pharmacological interventions remain inconclusive while there is inconsistent evidence for the efficacy of pharmacological treatment of this condition.^8^

This study aimed to identify the levels of apathy in Alzheimer’s disease, to identify the relationship between dementia severity and apathy, as well as to discuss the association of apathy with other psychopathological manifestations such as depression.

## Methods

### Patients

This study comprised 15 AD patients, both males and females, with an average age of 77 years and a mean level of formal educational of 4.9 years, living in the region of Rio Claro SP, Brazil. The physician responsible for the patients reached the diagnosis of Alzheimer’s disease and referred them for inclusion in this study. Patients maintained the routine medical recommendations and at the time of data collection were prescribed different medications by their respective physicians such as cholinesterase inhibitors, antidepressants, antipsychotics or benzodiazepines. The sociodemographic features of patients were as follows.

### Procedures

The diagnosis of AD was based on classical features, according to the consensus criteria for probable disease provided by the National Institute for Neurological and Communicative Disorders/Association (NINCDS/ADRDA),^9^ and the dementia assessment was performed according to the Diagnostic and Statistical Manual of Mental Disorders, 4th Revised Edition.^10^ The severity of dementia was initially classified using a semi-structured interview followed by the Clinical Dementia Rating (CDR) Scale.^11^ In the present study, the application of this scale was based on the Brazilian version in samples of dementia patients, as used by Maia et al.^12^ Trained raters evaluated patients using the Mini-Mental State Examination (MMSE),^13^ adapted to the Brazilian population by Brucki et al.,^14^ to identify global cognitive functioning, and the Clinical Dementia Rating was used to classify the severity levels of dementia. The raters also completed the Neuropsychiatric Inventory (NPI),^5^ considering global score and focusing on the apathy domain. Camozzato et al. ^15^ developed the reliability of the Brazilian version of NPI used in our study. Cummings et al.^5^ developed the NPI concerning psychopathological features in dementia. This scale assesses a wide range of behaviors common in dementia syndromes. In addition, to assess the level of apathy syndrome raters applied the Apathy Evaluation Scale (AES)^16^ the Brazilian version of which was prepared by Guimarães et al.^17^ The Global Deterioration Scale (GDS) was employed to characterize the profile of dementia severity,^18^ an instrument extensively applied to patients at academic and clinical sites throughout Brazil. To assess the level of depressive symptoms, the Cornell Scale for Depression in Dementia^19^ was applied using the Brazilian version from Carthery-Goulart et al.^20^

**Table 1:** Sociodemographic characteristics of patients with Alzheimer's disease.

Sociodemographic characteristics	Number of patients, mean, standard deviation, and range
Patients Females Males	15 10 5
Age (years)	77 (9.1); 63 to 95
Educational level (years)	4.9 (4.0); 0 to 15

### Statistical analyses

Data processing and statistical analysis were performed using the SPSS 10.0 software package, with descriptive analyses displaying mean values and standard deviations. Give the nonparametric data and small sample, correlations among scores on the Apathy Evaluation Scale and scores of other instruments including the Mini-Mental State Examination, Global Deterioration Scale, Cornell Scale for Depression in Dementia, Neuropsychiatric Inventory (total score, apathy domain, and caregiver burden) were established by *Spearman’s correlation* coefficient, with significant level for statistical analyses defined as p=0.05. For comparisons, the *Kruskal-Wallis* test was applied followed by *Bonferroni* adjustment and the Mann-Whitney U test. The level of significance was defined as p=0.01.

## Results

**Figure 1:**
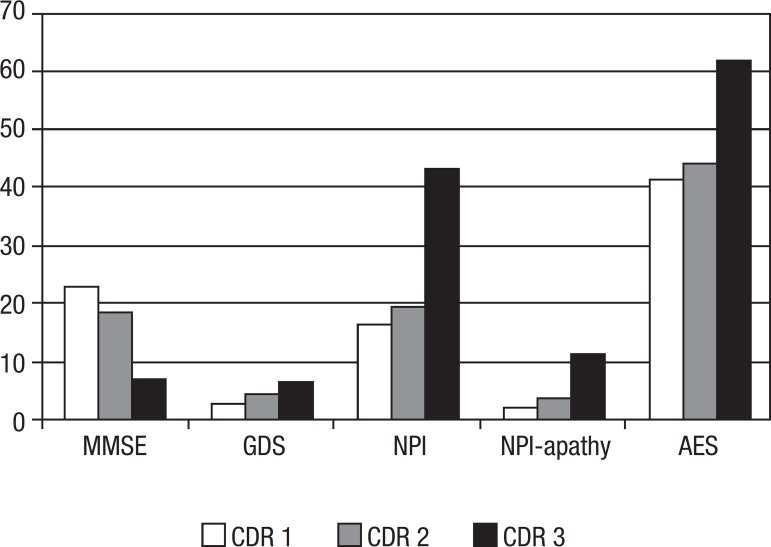
Cognitive impairment, global deterioration, and apathy among patients with Alzheimer’s disease by dementia severity.

According to the assessments applied, patients with clinically-advanced AD classified as CDR 3 presented greater cognitive impairment (MMSE), clinical global deterioration (GDS), higher levels of psychopathological symptoms (NPI total score), as well as more intensive apathetic syndrome (apathy domain of NPI and AES) than did subjects with mild (CDR 1) and moderate (CDR 2) dementia. These data are shown in Figure 1.

The correlations obtained by the Spearman coefficient showed association of apathy (measured by Apathy Evaluation Scale and NPI-Apathy domain) with cognitive impairment (measured by Mini-Mental State Examination), dementia severity (measured by Global Deterioration Scale) and depressive symptoms (measured by Cornell Scale for Depression in Dementia). These results are illustrated in Table 2.

**Table 2:** Association of apathy with other clinical features (Spearman's correlation coefficient).

Clinical features	Spearman coefficient: rho	p-value
MMSE × AES	-0.63	0.01
MMSE × NPI-Apathy domain	-0.81	0.01
GDS × AES	-0.58	0.02
GDS × NPI-Apathy domain	-0.80	0.01
Cornell Depression × AES	0.49	0.06
Cornell Depression × NPI-apathy	0.57	0.02

GDS: Global Deterioration Scale; MMSE: Mini-Mental State Examination; AES: Apathy Evaluation Scale; NPI-Apathy: Neuropsychiatric Inventory apathy domain; Cornell Depression: Cornell Scale for Depression in Dementia.

The *Kruskal-Wallis* analyses revealed differences between patients from CDR groups for the Mini-Mental State Examination (p=0.01), Global Deterioration Scale (p=0.01), and NPI-apathy domain (p=0.01). The *Mann-Whitney* U test showed marginal difference between patients classified as having mild (CDR 1) and moderate (CDR 2) dementia according to the Global Deterioration Scale (p=0.02). However, the *Mann-Whitney* U test detected significant differences between CDR 2 and CDR 3 patients on the Mini-Mental State Examination (p=0.01), Global Deterioration Scale (p<0.01), and NPI-apathy domain (p<0.01). Finally, comparison of CDR 1 and CDR 3 patients found a significant difference between the two groups on the Mini-Mental State Examination (p=0.01), Global Deterioration Scale (p<0.01), and in apathy from the NPI-apathy domain (p<0.01). Table 3 depicts these results.

**Table 3:** Clinical features related to scales (mean and standard deviation) by dementia severity.

Dementia severity	Number of patients	GDS	MMSE	NPI: total score	NPI - apathy domain	AES	Cornell depression
CDR 1	6	3±1^a^	23±3.3	16.5±20.6	2.3±3.2	41.5±18.4	8.66±6.34
CDR 2	5	4.4±0.5	18.6±2.5	19.6±17.5	3.8±4.7	44.4±17	8.40±5.12
CDR 3	4	6.5±0.6^b.c^	7±4.2^b.c^	43.2±15.4	11.3±1.5^b.c^	62±7.7	13.25±6.29

CDR: Clinical Dementia Rating; MMSE: Mini-Mental State Examination; NPI: Neuropsychiatric Inventory; GDS: Global Deterioration Scale; AES: Apathy Evaluation Scale.

a Significant difference between CDR1 and CDR2 patients;

b Significant difference between CDR2 and CDR3 patients;

c Significant difference between CDR1 and CDR3 patients.

As summarized in Table 3, the comparison among the three groups showed that patients classified into CDR 3 had significantly worse global deterioration than did those belonging to CDR 1 and CDR 2. Similarly, patients rated as CDR 2 also showed worse global deterioration than those rated as CDR 1. In addition, patients from the CDR 3 group revealed significantly greater impairment than those from CDR 1 and CDR 2 groups on the Mini-Mental State Examination and NPI-apathy domain.

## Discussion

Based on CDR severity levels, patients presented distinct decrease in cognition, global deterioration and apathy. These results suggest that both instruments - the Apathy Evaluation Scale^16^ and NPI apathy domain^5^ - were able to assess apathetic symptoms in patients with Alzheimer’s disease.

Unsurprisingly, patients with Alzheimer’s disease classified with more severe dementia presented high levels of apathy, a syndrome related to acute cognitive impairment and global deterioration.

In general, apathy is a persistent condition in Alzheimer’s disease patients, usually associated with dementia severity. This phenomenon predicts rapid cognitive and functional decline, including executive dysfunction and poor initiative, as well as global deterioration.^21^

In the present work, a significant association was found between clinical conditions (measured by MMSE and Global Deterioration Scale) and apathy levels measured by the NPI, confirming results reported by Turrió-Garriga et al.^22^ Our data were in agreement with reports by Starkstein et al.^23^ describing significant correlation of apathy with more accentuated cognitive decline and more severe deficits in global functionality. Concerning the association between dementia and apathy, these authors suggested in an earlier report that this syndrome was a neuropsychiatric marker of faster progression of clinical decline in Alzheimer’s disease.

Furthermore, we observed a relationship between global psychopathological manifestations based on NPI total score and apathetic symptoms on the Apathy Evaluation Scale. However, according to Levy et al.,^24^ there is no correlation between apathy and depression in patients with neurodegenerative diseases. However, other studies have demonstrated significant association between the two conditions and suggested that apathy is a significant predictor of depression.^21,25^ In our study, the statistical analysis revealed a correlation between apathy and depression in Alzheimer’s disease, measured by the Apathy Evaluation Scale and Cornell Scale for Depression in Dementia (marginally p=0.06), respectively, and using the NPI-apathy domain and Cornell for Depression in Dementia (p=0.02). If the sample were composed of a greater number of patients, the statistical analysis may indicate a significant correlation between the first clinical conditions (MMSE).

In Alzheimer’s disease, establishing a clear cut difference between apathy and depression continues to pose a challenge because of overlapping prediction of psychopathological features in both conditions, such as reduced volition, loss of interest and psychomotor retardation.^21^ Despite these difficulties, there is a growing trend toward accepting apathy and depression as two distinct syndromes. For instance, several dysphoric mood symptoms such as guilty feelings and anhedonia are prominent psychopathological features of depression, while lack of emotional responsiveness and absence of motivation characterize the apathy syndrome.^6^

Concerning neurobiological connections, dysfunctions of frontal regions of the brain have been implicated in apathy syndrome. According to Levy & Dubois,^26^ apathy should be understood as a heterogeneous disorder resulting from at least three phenomena related to the topography of prefrontal cortex and basal ganglia. The first describes the affective-emotional process and involves the ventromedial prefrontal cortex. This circuit integrates affective or emotional value to behavior. The second constitutes the cognitive process and is regulated by the dorsolateral prefrontal cortex and caudate nuclei. Essentially, this circuit is responsible for executive elaboration and goal-directed behaviors. The third phenomena relates to the self-activation cognitive process and has been observed in more severe forms of apathy, characterized by difficulty in self-initiated actions and thoughts. This behavior can be observed after bilateral lesions in internal portions of the *pallidum*,^27^ or after extensive lesions of the prefrontal medial wall.^28^

Holthoff et al.^25^ suggest that in early Alzheimer’s disease it is possible to first detect functional deficits in the orbitofrontal areas, since left orbitofrontal structures have revealed hypometabolism in Alzheimer’s disease patients with apathy in comparison with patients without this syndrome. These authors explain that these brain regions represent a convergence zone for exteroceptive sensory inputs from association cortices and interoceptive inputs from limbic structures linked to emotional processing and cognition. It is plausible that emotional and cognitive processes contribute to the modulation of motivational behaviors. In addition, anterior cingulated gyrus and frontal-subcortical circuits have been involved in apathy symptoms, such that this structure mediates the connection among emotion, cognition, drive, and motor control.^29^ Studies have verified hypoperfusion and hypometabolism of the anterior cingulate gyrus and related frontosubcortical pathways as the most frequent neurobiological components associated with apathy in Alzheimer’s disease.^6,30^ According to several authors,^6,31^ the cingulate integrates the neurobiological systems which govern goal-directed behavior and represents a convergence zone for the cortico-subcortical pathways responsible for frontal processing, including executive control and attention as well as vegetative, sensory and reward working areas of the brain.

According to Esposito et al.,^32^ apathy is correlated with executive deficits and akin to depression, this phenomenon is a predictor of lack of initiative in Alzheimer’s disease.

To better the understanding of the neuropsychiatric manifestations of apathy in Alzheimer’s disease, Guimarães et al.^33^ revised the literature and proposed a pathophysiological model combining results from several procedures such as neuroimaging, neuropathology and experimental investigations. These authors emphasized mainly dysfunction of orbitofrontal cortex, anterior cingulate gyrus, basal ganglia and dopaminergic system as brain processes involved in reduced decision-making and decreased activation of goal-directed behaviors - phenomena linked to disturbances that generate and modulate voluntary performance in apathy syndrome.

The main limitations of this study included the small sample size, absence of a control group to compare results, and the cross-sectional nature of the investigation. In addition, drug prescriptions such as antipsychotics or benzodiazepines likely interfered with a precise identification of apathy and other neuropsychiatric disturbances. Given these conditions, this data is unlikely to be generalized. Despite these limitations, we sought to address a controversial subject whose relevance among research centers is growing.

In conclusion, our results showed that apathy is a relevant syndrome in patients with Alzheimer’s disease and increases with global deterioration. Furthermore, although apathy and depression can be considered distinct syndromes, their clinical psychopathological manifestations are related. The debate over whether an improvement in apathy syndrome attenuates global deterioration and reduces cognitive and functional decline, remains a pertinent question which warrants further clarification.
